# Blockchain-enabled access control to prevent cyber attacks in IoT: Systematic literature review

**DOI:** 10.3389/fdata.2022.1081770

**Published:** 2023-01-12

**Authors:** Rinki Singh, Deepika Kukreja, Deepak Kumar Sharma

**Affiliations:** ^1^Department of Information Technology, Netaji Subhas University of Technology, New Delhi, India; ^2^Department of Information Technology, Indira Gandhi Delhi Technical University for Women, New Delhi, India

**Keywords:** cyber attacks, Internet of Things, security, privacy, blockchain, access control, smart contract

## Abstract

Internet of Things (IoT) enables communication among objects to collect information and make decisions to improve the quality of life. There are several unresolved security and privacy concerns in IoT due to multiple resource constrained devices, which lead to various cyber attacks. The conventional access control techniques depend on a central authority that further poses privacy and scalability issues in IoT. Various problems with access control in IoT can be resolved to prevent various cyber attacks using the decentralization and immutability properties of the blockchain. This study explored the current research trends in blockchain-enabled secure access control mechanisms and also identifies their applicability in creating reliable access control solutions for IoT. The basic properties of blockchain, such as decentralization, auditability, transparency, and immutability, act as the propulsion that provides integrity and security, disregarding the participation of an external entity. Initially, the application of blockchain was created only for cryptocurrencies but with the introduction of Ethereum, which allows the writiting and execution of smart contracts, applications other than cryptocurrencies are also being created. As various research articles have been written on the usage of different types of blockchains for creating secure access control solutions for IoT, this study intends to find and examine such primary researches as well as come up with a systematic review of various findings. This study perceives the most frequently utilized blockchain for creating blockchain-based access control solutions to prevent various cyber attacks and also discusses the improvement in access control mechanisms using blockchain along with smart contracts in IoT. The present study also discusses the obstacles in building decentralized access control solutions for IoT systems as well as future research areas. For new researchers, this article is a nice place to start and a strong reference point.

## 1. Introduction

Internet of Things (IoT) has changed the way people interact and communicate with each other. Due to the resource constrained property of IoT devices, access control is one of the primary challenges that IoT is confronted with, which further leads to various cyber attacks. The majority of existing access control techniques for IoT rely on a central authority that makes these techniques prone to different types of threats. The potential of blockchain to provide privacy, integrity, and security without depending on a third party makes it the best contender for providing secure access control in IoT to prevent various cyber attacks. However, the blockchain, which was initially presented in 2008 by Satoshi Nakamoto as the key technology for Bitcoin (Nakamoto, 2008), did not gain widespread use outside of cryptocurrencies, until the release of Ethereum (Buterin, 2014). Ethereum allows the creation and execution of smart contracts to specify the criteria and rules, in the form of a code, to which all parties involved in the deal have to agree. Activities mentioned in the contract may be carried out only if the conditions and rules are satisfied. Currently, Ethereum, Hyperledger Fabric (Androulaki et al., 2018), and many other blockchain platforms support the execution of smart contracts. Blockchain with smart contracts can be applied to the development of various applications, such as smart agriculture, smart grids, smart cities (Hakak et al., 2020), and many more. To find answers to various research issues and generate new paths, the present study identified and critically examined the current research explicitly relevant to the blockchain-enabled safe access management in IoT. The Systematic Literature Review (SLR) is organized as follows: Related work is mentioned in Section Related work and the procedure for the review process is described in Section Research methodology. Summary drafted after a thorough analysis of the collected studies is presented in Section Findings. The answers to various research questions, included in this study, are addressed in Section Answers to research questions. Various challenges and directions for future work with respect to the blockchain-enabled secure access control in IoT are given in Section Challenges and future work. Concluding remarks are presented in Section Limitations of using blockchain in access control for IoT.

## 2. Related work

As per our knowledge, no such SLR is available on the blockchain-enabled secure access control in IoT. However, Lone and Naaz (2021) performed an SLR that emphasized safeguarding the IoT and Internet using smart contracts. According to their findings, many of the security services can be achieved using blockchain smart contracts, some of which are non-repudiation, integrity, protection of data, secure access control, and authentication. Their research works also found that Ethereum, followed by Hyperledger Fabric, was a popular blockchain for creating smart contract-based security mechanisms. They suggested that, for the construction of security solutions for IoT, future research should focus on the requirement for enhanced and private smart contracts, as well as on a scalable and secure blockchain platform that facilitates the execution of smart contracts. Stojkov et al. (2020) conducted a study on traditional and blockchain-driven access control solutions in IoT to find out how challenges in conventional access control solutions can be overcome using the blockchain technology. It was concluded that it is possible to make the transition from traditional to blockchain-based solutions for various applications. Patil et al. (2021) performed a study on the blockchain-enabled existing security techniques in the areas of health care, IoT access control, supply chain, and Vehicular *Ad Hoc* Networks (VANETs). They evaluated existing solutions on the basis of storage and computation overhead, scalability, privacy, extensibility, and accuracy. They concluded that the consortium of blockchain combined with an effectual consensus algorithm serves as a better option for various applications. Dadhania and Patel (2020) presented a review on potential improvement in IoT access control with decentralized architecture using blockchain and concluded that IoT transactions can be made more secure using the potential of blockchain technology.

Butun and Osterberg (2021) conducted a study to find out the usability of traditional and blockchain-enabled access control mechanisms in an IoT environment with blockchain systems along with permissioned and permissionless blockchains. The authors concluded that the security of IoT networks can be enhanced using permissioned blockchains, and hence, this security feature would be more suited to an IoT environment than to an IoT environment with permissionless blockchains. They also provided a remedy to facilitate access control functionality in permissioned blockchains by focusing on the recent access control mechanisms suggested for peer-to-peer networks.

## 3. Research methodology

The SLR was conducted as per the guidelines presented by Kitchenham and Charters (2007) to explore the answers to the research questions. Many sources, including significant web databases, were investigated to get an unprejudiced and comprehensive viewpoint. The following databases were combed through:

- Elsevier,
- ACM (Association for Computing Machinery) Digital Library,
- MDPI (Multidisciplinary Digital Publishing Institute),
- Springer Link, and
- IEEE Xplore Digital Library.

To review extant publications, compiling the results and recapitulating the factual data referencing the utilization of blockchain for secure access control in IoT is the major goal of this study. To accomplish our objectives, the study considers the research questions as listed in Table 1.

**Table 1:** Research questions.

**Research questions**	**Significance**
RQ1: How many and what are the different blockchain platforms used to implement secure access control in IoT?	Recognize the most widely used blockchain platforms for developing secure access control solutions in IoT.
RQ2: How blockchain improves the strength of access control solutions in IoT?	Assess the efficacy and strength of blockchain enabled access control solutions.
RQ3: How convincing is a smart contract's appropriateness for solving access control concerns in IoT?	Find out the practical applicability of smart contract-enabled access control solutions in addressing access control concerns in IoT.

### 3.1. Selection of primary studies

Selected databases were searched using specific words to obtain a collection of primary research. We were able to obtain a wide range of results using general search phrases. The main search words were entered between the logical AND and OR operators and expressed as (“Distributed ledger” OR “Blockchain”) AND (“IOT” OR “Internet of Things”) AND (“Access Control”). The searches were undertaken in 9 August 2022, and publications from 2017 to the aforementioned date were examined. The results of a search query applied to several databases were subjected to a filtering procedure. We acquired a set of primary studies by applying the inclusion–exclusion criteria (presented in Section Inclusion–exclusion criteria) to the results received from a previous step.

### 3.2. Inclusion–exclusion criteria

These inclusion–exclusion criteria are established to make sure the selected publications accommodate with our SLR. The key inclusion–exclusion measures are presented in Table 2.

**Table 2:** Inclusion–exclusion criteria.

**Inclusion criteria**	**Exclusion criteria**
1. Publications belonging to the area of blockchain-enabled access control in IoT?	1. Publications that does not belong to the area of blockchain-enabled access control in IoT.
2. Publications that present data related to blockchains used and access control in IoT.	2. Survey or a review paper.
3. The article presented at a conference or journal.	3. The paper not presented at a conference or a journal, like blog posts, white papers, government documents.
4. Paper published in the English language.	4. Paper published in a language other than English.

### 3.3. Selection results and quality assessment

**Figure 1:**
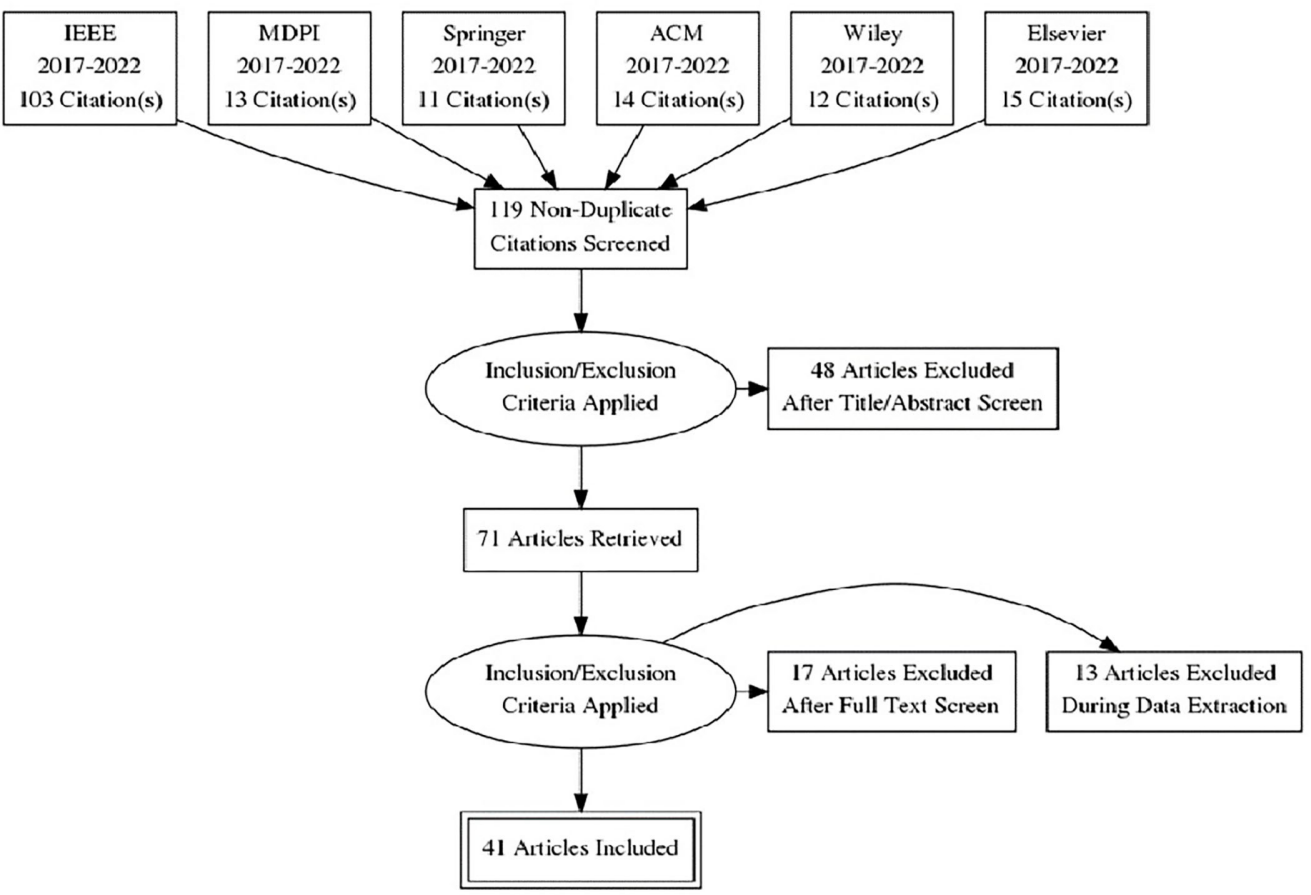
PRISMA diagram of selection process.

Figure 1 represents the Preferred Reporting Items for Systematic Reviews and Meta-Analyses (PRISMA) diagram of the selection process. A total of 168 papers were retrieved using predefined search criteria, out of which 49 were discovered to be duplicates, resulting in 119 non-identical publications. The number of publications shrank from 119 to 71 after applying the inclusion–exclusion measures. Seventy-one articles were reviewed in their entirety, and the inclusion–exclusion criteria were applied again, leaving 41 articles for SLR evaluation.

### 3.4. Publications over time

**Figure 2:**
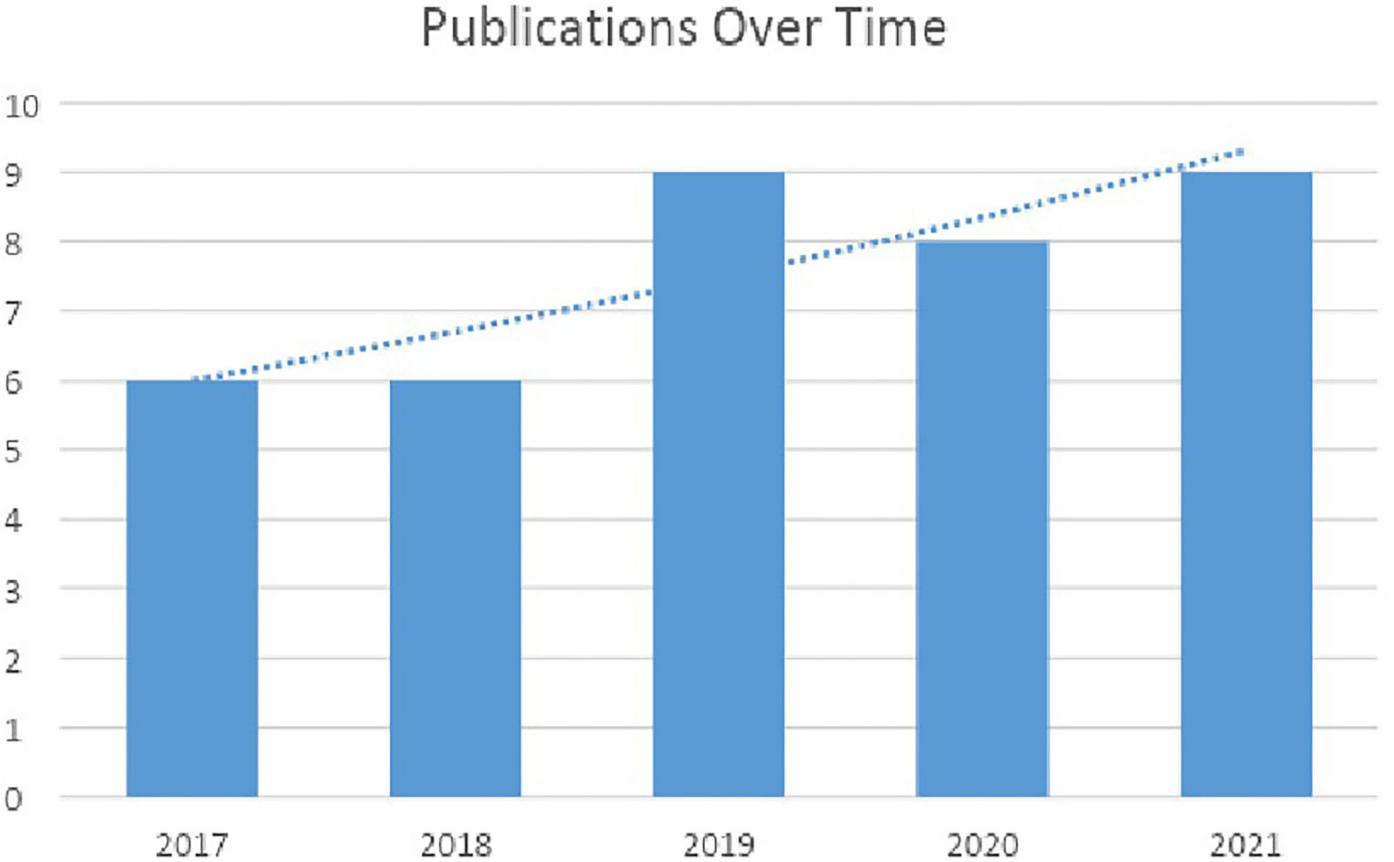
Number of publications over time.

Despite the notion of blockchain having been around since the introduction of Bitcoin in late 2008, there were very few publications available before the introduction of Ethereum. The number of final primary researches published in the years 2017 until 2021 is shown in Figure 2.

## 4. Findings

**Figure 3:**
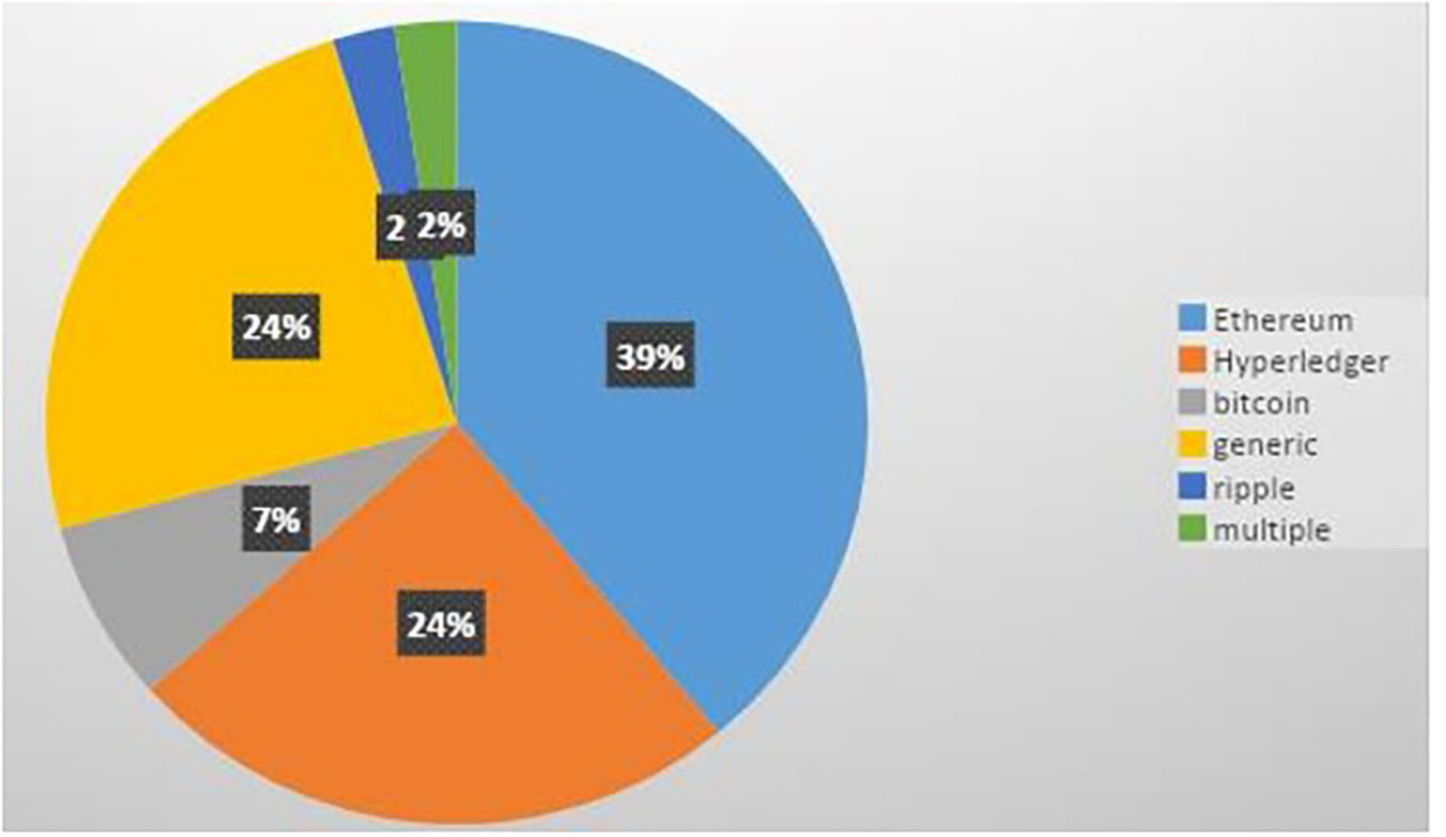
Blockchain platform used for access control solutions in IoT.

Every publication from the final list was reviewed, and important findings were retrieved and are summarized in Table 3 after a comprehensive evaluation. The present study categorized the studies on the basis of blockchain platform they had used to provide access control in IoT. Figure 3 presents the classification of different blockchain platforms used for implementing secure access control in IoT, as identified by this SLR. Most of the primary research focused on the Ethereum (39%) and Hyperledger Fabric (24%) blockchain for secure access control. Other primary studies explored Bitcoin (7%) or some other blockchains such as Ripple (3%). Some studies have not mentioned the name of the blockchain used but specified some of the features for the same (generic). Blockchains have played a crucial role in dealing with the access control issues in all primary studies.

**Table 3:** Key findings and themes of primary studies.

**S.no**	**Publication**	**Blockchain used**	**Smart contract used**	**Key findings**
1	Ouaddah et al. (2017b)	Ethereum	Yes	Presented the possibility of using second-generation blockchain to create an enhanced version of the distributed access control architecture.
2	Xu et al. (2018)	Ethereum	Yes	Presented a blockchain-based distributed, capability-enabled access control framework to handle access control challenges in IoT.
3	Novo (2018)	Ethereum	Yes	Proposed a fully distributed, blockchain-based framework for the arbitration of roles and authorization in the Internet of Things.
4	Ourad et al. (2018)	Ethereum	Yes	Proposed a solution for authenticated and secure communication among IOT devices, using blockchain.
5	Yutaka et al. (2019)	Ethereum	Yes	A system based on blockchain, and the Attribute Based Access Control (ABAC) model is proposed to implement a distributed and reliable access control for IoT.
6	Novo (2019))	Ethereum	Yes	An architecture for IoT access management was presented, in which the credentials and authorization to access various resources are kept on the blockchain.
7	Wang et al. (2019)	Ethereum	Yes	Proposed a blockchain-enabled Attribute-Based Decentralized Access Control Solution for IoT.
8	Breiki et al. (2019)	Ethereum	Yes	Blockchain and trusted oracles are used to implement distributed access management framework for IoT.
9	Putra et al. (2019)	Ethereum	Yes	Proposed a blockchain-enabled access control framework for IoT.
10	Tapas et al. (2020)	Ethereum	Yes	Presented a blockchain-enabled model, focused to observe access control and delegation mechanism in Internet of Things.
11	Sultana et al. (2020)	Ethereum	Yes	Proposed a blockchain-enabled mechanism for reliable access control and effective communication among IoT devices.
12	Yu et al. (2020)	Ethereum	Yes	Proposed an access management solution using blockchain, compatible with the attribute based encryption technique.
13	Oktian and Lee (2021)	Ethereum	Yes	A blockchain enabled access control architecture for resource constrained IoT devices is proposed.
14	Putra et al. (2021)	Ethereum	Yes	Designed a blockchain enabled ABAC solution for IoT devices having an additional Trust and Reputation System.
15	Liu et al. (2021)	Ethereum	Yes	Presented a blockchain and distributed identifier-based architecture for capability-based access control to resolve identification and access control issues in IoT devices.
16	Xiang and Yuanyuan (2021)	Ethereum	Yes	Proposed a blockchain-enabled mechanism to resolve the scalability issue of access management in IoT.
17	Li et al. (2021)	Fisco Bcos and Ethereum	Yes	Proposed a double-layer blockchain enabled access control framework to minimize the communication overhead for IoT devices.
18	Islam and Madria (2019)	Hyperledger	Yes	Implemented an ABAC on a permissioned blockchain for distributed access control in IoT.
19	Ali et al. (2019)	Hyperledger	Yes	Presented a blockchain-enabled hybrid framework for permission delegation (event-based and query-based) and access control in IoT
20	Ding et al. (2019)	Hyperledger	-	Proposed ABAC for IoT where blockchain is used to resolve issues like a single point of failure and data tampering.
21	Liu et al. (2020)	Hyperledger	Yes	Designed a dynamic and decentralized ABAC using blockchain for IoT.
22	Xu et al. (2020)	Hyperledger	Yes	Proposed a distributed attribute-based hierarchical encryption using multi-level authorization to implement fine-grained access control.
23	Zhang et al. (2020)	Hyperledger	Yes	Proposed a blockchain based ABAC framework which provide distributed, malleable, and fine-grained authorization for Internet of Things.
24	Iftekhar et al. (2021)	Hyperledger	Yes	Created a blockchain-enabled access control system that uses rules and programmatic access management to make groups of people and devices in IoT.
25	Sun et al. (2021)	Hyperledger	Yes	A reliable, lightweight, and cross- functional access control system was built for IoT by integrating an Identity-Based Signature, permissioned blockchain and ABAC.
26	Han et al. (2022)	Hyperledger	Yes	Proposed a blockchain-enabled auditable access control solution, confirming the security of personal data in the Internet of Things.
27	Li T. et al. (2022)	Hyperledger	Yes	Constructed a blockchain-enabled privacy-preserving private data exchanging scheme for IoT.
28	Bera et al. (2020)	Ripple	No	Blockchain-based access control mechanism that provide secure communication between drones and the Ground Station Server as well as secure inter drone communication along with the resistance from various attacks.
29	Outchakoucht et al. (2017)	Generic	Yes	A blockchain enabled, dynamic and fully decentralized access control mechanism for IoT was proposed.
30	Ouaddah et al. (2017c)	Generic	Yes	Proposed a decentralized, privacy-preserving, and authorization control architecture where access control was performed by utilizing the consistency of blockchain
31	Hwang et al. (2018)	Generic	Yes	A dynamic access control strategy was proposed to address the issues with existing access control techniques in IoT.
32	El Kalam et al. (2018)	Generic	Yes	Blockchain and Reinforcement Learning tools, provide an auto-corrected and dynamic security policy having full control over IoT devices
33	Dukkipati et al. (2018)	Generic	Yes	Provide a blockchain enabled access management solution for IoT that helps to control the access of data.
34	Ma et al. (2019)	Generic	-	Blockchain and fog computing are used to implement distributed key management architecture to provide cross-domain access.
35	Saha et al. (2020)	Generic	-	A blockchain-enabled access control mechanism was established for securely exchanging private and confidential data.
36	Algarni et al. (2021)	Generic	-	Proposed a blockchain enabled solution making use of multi-agent system to produce a distributed access control that provide reliable communication among IoT devices, fog nodes and cloud server.
37	Bera et al. (2021)	Generic	-	A blockchain enabled distributed access control solution was proposed for smart-grid system to transfer the data securely from smart meters to the corresponding service providers.
38	Ahmed et al. (2022)	Generic	-	A Blockchain based authentication framework to reduce the computational load by organizing IoT devices into “clusters”.
39	Ouaddah et al. (2017a)	Bitcoin	Yes	Blockchain was employed as a decentralized access control manager to provide a decentralized privacy-preserving authorization management system.
40	Pinno et al. (2017)	Bitcoin	No	Presented a blockchain-based mechanism using multiple blockchains to deal with different issues associated with access control in IoT.
41	Shafagh et al. (2017)	Bitcoin	No	Blockchain was employed as a decentralized access control layer to implement a safe and adaptable access control mechanism.

## 5. Answers to research questions

Blockchains along with Turing-complete smart contracts enable us to conduct increasingly complicated activities in a variety of sectors, with endless applications. As blockchains cater to several purposes, the researchers used various blockchain platforms, which are delineated in the later part of this section. This study perceives the most frequently utilized blockchain for creating blockchain-based access control solutions to prevent various cyber attacks and also discusses the improvement in access control mechanisms by using blockchain along with smart contracts in IoT. Characteristics of a blockchain, such as traceability, decentralization, and robustness, are intrinsic by nature. When the number of participating nodes is large, decentralization and confidence in individual nodes are prevalent, which increase the blockchain security and dependability. However, based on our preliminary research, we conclude that the access control in IoT can be enhanced further by utilizing the bespoke property of smart contracts.

### 5.1. RQ1: How many and what are the different blockchain platforms used to implement secure access control in IoT?

As per our findings, a substantial number of primary studies (Ouaddah et al., 2017b; Novo, 2018, 2019; Ourad et al., 2018; Xu et al., 2018; Breiki et al., 2019; Putra et al., 2019, 2021; Wang et al., 2019; Yutaka et al., 2019; Sultana et al., 2020; Tapas et al., 2020; Yu et al., 2020; Liu et al., 2021 and Oktian and Lee, 2021; Xiang and Yuanyuan, 2021) have used Ethereum platform for developing blockchain-driven access control mechanisms in Internet of Things (IoT). Some other studies (Ali et al., 2019; Ding et al., 2019; Islam and Madria, 2019; Liu et al., 2020; Xu et al., 2020; Zhang et al., 2020; Iftekhar et al., 2021; Sun et al., 2021; Han et al., 2022; Li T. et al., 2022) have made use of the Hyperledger Fabric platform to deal with access control issues in IoT. Primary studies (Bera et al., 2020) utilized the Ripple Blockchain platform for secure access control in Internet of Drones (IoD). There are some other studies (Ouaddah et al., 2017c; Outchakoucht et al., 2017; Dukkipati et al., 2018; El Kalam et al., 2018; Hwang et al., 2018; Ma et al., 2019; Saha et al., 2020; Algarni et al., 2021; Bera et al., 2021; Ahmed et al., 2022) where the researchers have exploited customized Blockchain platforms for access control solutions in IoT. Furthermore, primary studies (Li et al., 2021) addressed secure (Kukreja et al., 2019a,b) access control in IoT using multiple blockchains (Ethereum, FISCO Bcos). Some studies (Ouaddah et al., 2017c; Outchakoucht et al., 2017; Dukkipati et al., 2018; El Kalam et al., 2018; Hwang et al., 2018) offered smart contract-based access control solutions that can be applied on a generic blockchain having smart contracts. Ouaddah et al. (2017a), Pinno et al. (2017), and Shafagh et al. (2017) made use of Bitcoin platform for designing access control solutions in IoT.

### 5.2. RQ2: How does blockchain improve the strength of access control solutions in IoT?

Single point of failure, lack of scalability, and privacy are some of the problems that one comes across in conventional access control techniques that work under a centralized entity. Based on primary studies shown in Table 3, we observed that blockchain-based access control mechanisms do not require a remarkable difference to the present network architecture, but they rely on the blockchain's intrinsic attributes and the substantial programming attributes of smart contracts. All primary researches depend on the transparent, decentralized, tamperproof, and traceable properties of the blockchain technology. Blockchain's decentralized and immutable nature can aid in overcoming access control concerns, as some of the studies used these properties (Li D. et al., 2022; Tao et al., 2022) as well as the configurable nature of smart contracts for creating access control solutions in IoT. Furthermore, primary research has used blockchain's tamper-resistant characteristics to ensure data integrity in access control systems.

### 5.3. RQ3: How convincing is smart contract's appropriateness for solving access control concerns in IoT?

Like blockchain, smart contracts also cannot guarantee to resolve access control issues in IoT, but they can support the extant technical solutions to resolve these issues. As the blockchain uses its fundamental properties such as traceability, immutability, and transparency, smart contracts also take advantage of its adaptable features such as customizability, resemblance to commonly used scripting languages, and turing-completeness. According to the majority of primary researches, the smart contracts with existing architecture provide the answers to many access control issues in IoT. Apart from reducing the requirement for a change in the architecture of existing networks, smart contract also allows them to be changed if required to intensify the IoT framework. Most of the articles mentioned in Table 3 give substantial proof that smart contracts may have their usability to solve access control challenges in IoT environment, either in a standalone way or in combination with other technologies.

## 6. Challenges and future work

The blockchain technology offers a few concerns and constraints. Some of the fundamental concerns of blockchain-enabled access control are addressed here. As per the findings, Ethereum is the most frequently utilized blockchain by researchers for building blockchain-enabled access control mechanisms. One of the most common features of Ethereum smart contracts is that that they cannot be amended or updated after being deployed on a blockchain network. This feature presents both advantages and disadvantages. On the plus side, the platform is reliable because once the smart contracts are implemented, they cannot be amended to deceive someone or obtain unlawful benefit. On the minus side, the platform is not upgradable, that is, it cannot respond to progressive changes as fixing some issue in a previously implemented smart contract. With millions of connected IoT devices, achieving a high transaction throughput and low latency requirements is another major challenge. Furthermore, the present study observed that the majority of researchers suggested smart contract-enabled secure access control solutions for IoT. Another major concern is the expanding dimensions of blockchain over time. Since every transaction incurs a storage cost, blockchain grows in dimensions with each access/authentication request, which may restrict its scalability in accomplishing the needs of specific IoT applications. Resolving these difficulties and evaluating the suggested blockchain-based access control methods are therefore left as future work to be resolved. The research directions to achieve secure access control in IoT using blockchain lead to Cyber-Security Analysis (understanding the system behavior from the cyber-security perspective) and Performance Analysis (end-to-end performance analysis of the underlying blockchain).

Focusing on the findings of SLR, it was observed that, to achieve secure access control in an IoT network, a secure, lightweight, and scalable blockchain platform having upgradable and private smart contracts is required in the future.

## 7. Limitations of using blockchain in access control for IoT

Blockchains along with smart contracts integrated with IoT enable us to conduct increasingly complicated activities in a variety of sectors, with nearly endless applications. However, the blockchain technology has few concerns that should be investigated further:

- IoT devices are battery powered with low-energy requirements. However, with the integration of blockchain, the energy requirements of devices need to be explored further.
- As the number of nodes in IoT increases, blockchain scales poorly, which should be addressed at the earliest.
- Vulnerabilities, such as DoS attacks and the 51% attack, are common with blockchain and must be handled with utmost care.

## 8. Conclusion

From this SLR, it can be concluded that Hyperledger Fabric is the second most frequently utilized blockchain for creating blockchain-based access control solutions to prevent various cyber attacks, whereas Ethereum is the number one pick. The present study also discusses the improvement in access control mechanisms using blockchain along with smart contracts in IoT and difficulties that are currently preventing the use of blockchain in IoT.

## Data availability statement

The original contributions presented in the study are included in the article/supplementary material, further inquiries can be directed to the corresponding author.

## Author contributions

All authors listed have made a substantial, direct, and intellectual contribution to the work and approved it for publication.
